# Supplementary material for: A universal system for boosting gene expression in eukaryotic cell-lines
Source: Nat Commun. 2024 Mar 16;15:2394. doi: 10.1038/s41467-024-46573-5 (PMC10944472; doi:10.1038/s41467-024-46573-5)
Supplement: Supplementary file 1 — Supplementary Information [file 41467_2024_46573_MOESM1_ESM.pdf]

Supplementary Information for:

A universal system for boosting gene expression in Eukaryotic cell-lines

Inbal Vaknin[1], Or Wilinger[1], Jonathan Mandl[4], Hadar Heuberger[3], Dan Ben-Ami[3], Yi Zeng[1], Sarah Goldberg[1], Yaron Orenstein[4,5], and Roee Amit[1,2]

[1]Department of Biotechnology and Food Engineering, Technion, Haifa, Israel

[2]The Russell Berrie Nanotechnology Institute, Technion, Haifa, Israel

[3]School of Electrical and Computer Engineering, Ben-Gurion University of the Negev

[4]Department of Computer Science, Bar-Ilan University, Ramat-Gan, Israel

[5]The Mina and Everard Goodman Faculty of Life Sciences, Bar-Ilan University, Ramat-Gan, Israel

Supplemental Methods

Growth Curve Analysis

We grew the 43 validation variants in SD-Ura + 2% glucose and tracked their growth via OD measurement as function of time (Supplemental Figure 7a - circles). For each strain we then fitted the OD measurements (Supplemental Figure 7a – blue line) with the following model for exponential growth:

$$OD_{600}(t) = C + \frac{L}{(1 + e^{-k(t-t_0)})},$$

Where C is background OD levels, L is the max OD, k growth rate, and t_0 corresponds to the lag time (i.e. time at which the culture reaches OD of L/2). Using this model we extracted the growth rate for each strain and for both repeats. We plot in Supplemental Figure 7b the different fitted growth rates (k) for both repeats that were measured for each variant. The results show that the growth rates for all experiments was found to be within a narrow range of ~0.4-1.3 (1/hr) without any significant correlation between duplicates.

Supplemental Data Files

Supplementary Data 1: Synthetic URSs OL design (excel file).

Supplementary Data 2: Raw NGS raw data (excel file).

Supplementary Data 3: MBO model predictions based on 2098 variants (excel file).

Supplementary Data 4: Summary of the mHG p-values of all the 41 motifs (excel file).

Supplementary Data 5: Summary of the mHG p-values of all the 20 motifs containing K and/or M (excel file).

Supplementary Data 6: Variants used in validation experiments in yeast cells and CHO cells (excel file).

Supplementary Data 7: Pearson correlation data for the validation sets (excel file)

Supplemental Figures

#motif					Sequence used for OL	Regulatory function
1	bZip	bZip	CST6		ATGACGKCMT	CST6-activating bZip- dual function
2	Lag1	RBPJ		cbf11/12	GCGTGGGAA	Repressing
3		bHLH	bHLH	bHLH	GTCACGTGAC	Dual function
4	SP1/KLF				MCMCGCCCA	Dual function
5			Rap1		AMACCCACACMCC	Dual function
6				Rap1	ACCCAKACACC	Dual function
7	Ttk				TCCTGCAGGA	Repressing
8			YAP		GMTTACGTMAKC	Dual function
9					ACAACAACAM	Unknown
10					GCTAAGCCGC	Unknown
11					ACGATCCTCA	Unknown
12					CTGACCTMCC	Unknown
13					GTTACCCTGC	Unknown
14					KMTAMGCCAC	Unknown
15					KAGGCGCAGC	Unknown
16					AACGAGGCKK	Unknown
17					CAGCAAAAAT	Unknown
18					CAGGTAACAA	Unknown
19					ATCGTACGAT	Unknown
20					GMTAMGCCAC	Unknown
21					ACCAMTCGGA	Unknown
22					MTGTCAATCA	Unknown

#motif					Sequence used for OL	Regulatory function
23		Nfi			KGGMACACTKCCM	Activating
24		Nrf1			GCGCATGCGC	Activating
25		NR			GGTCAAAGGTCA	Repressing
26		Ets			ACMGGAAGTG	Activating
27		Yy1/2			CGCCMTGTTG	Dual function
28			Reb1		KCMGGGTAAC	Dual function
29			Abf1		TCACTCACTACGA	Activating
30			STP		CGGCGCTAGC	Activating
31			Opi1		GGTTCGAACC	Repressing
32				Prz1	GCTGCGCCAC	Activating
33				Tbf1	ACCCTTACCCT	Repressing
34		Pou51			TATGCAAATK	Activating
35		AP2			TGCCTGAGGCA	Activating
36		Zic3			CCCCCCGCTG	Activating
37		NF-kB			GGGGAATCCCC	Dual function
38		KLF			ACCMCGCCCM	Dual function
39		Srf			CCATATATGG	Activating
40		Essrb			TTCAAGGTCA	Dual function
41		CTCF			MCGCCCCCTA	Dual function

Supplementary Figure 1: A List of all DNA motifs used in the sURSs OL.

For each of the 41 motifs, the source organism, sequence logo, sequence used in the OL and the regulatory function, are included. Organisms include the Drosophila S2 cells, mouse ES cells, S. cerevisiae and S. Pombe.

a

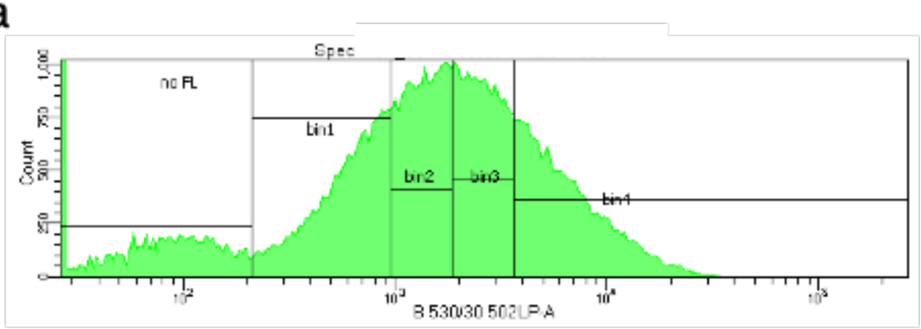

b

Population	#Events	%Parent	B 530/30 50... Min	B 530/30 50... Max	B 530/30 50... Mean	B 530/30 50... Median
All Events	100,000	####	-343	262,143	2,809	1,403
P1	85,795	85.8	-343	54,176	2,871	1,618
P2	78,976	92.1	-343	54,176	2,694	1,554
bin1	17,043	21.6	212	947	607	616
bin2	17,181	21.8	948	1,848	1,364	1,346
bin3	16,821	21.3	1,849	3,605	2,596	2,537
bin4	17,846	22.6	3,606	54,176	7,541	5,965
no FL	10,085	12.8	-343	211	69	69

c d e

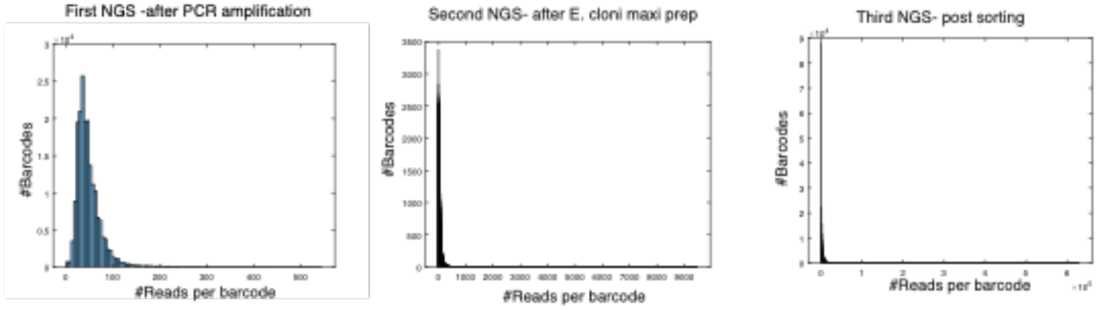

Supplementary Figure 2: FACS-generated data from the sorting experiment and Distribution plots for sURS OL's deep sequencing during the cloning stages.

(a) A fluorescence histogram showing the sorting experiment, the 4 sorted bins and the "No FL" gate, containing the non-fluorescent yeast population, determined by the wild type W303 yeast cells. (b) A table showing the different populations in the sorting experiment, number of events analyzed in the FACS, bin percentages (% parent), min and max FL values of each gate, and the mean and median values for each gated population. (c-e) The plots represent the frequency of barcodes and their respective read count. Read counts for every barcode. Overall, 3 NGS runs were performed: (c) After the first PCR amplification of the OL. 189,495 barcodes were retrieved, out of 189,990 barcodes in total. (d) After the cloning and the plasmids' extractions from the E. cloni

bacteria. 183,401 barcodes were retrieved. (e) Post-sorting the OL-integrated yeast cells into 4 bins. 149,336 barcodes were retrieved. Source data for panels c-e are provided as a Source Data file.

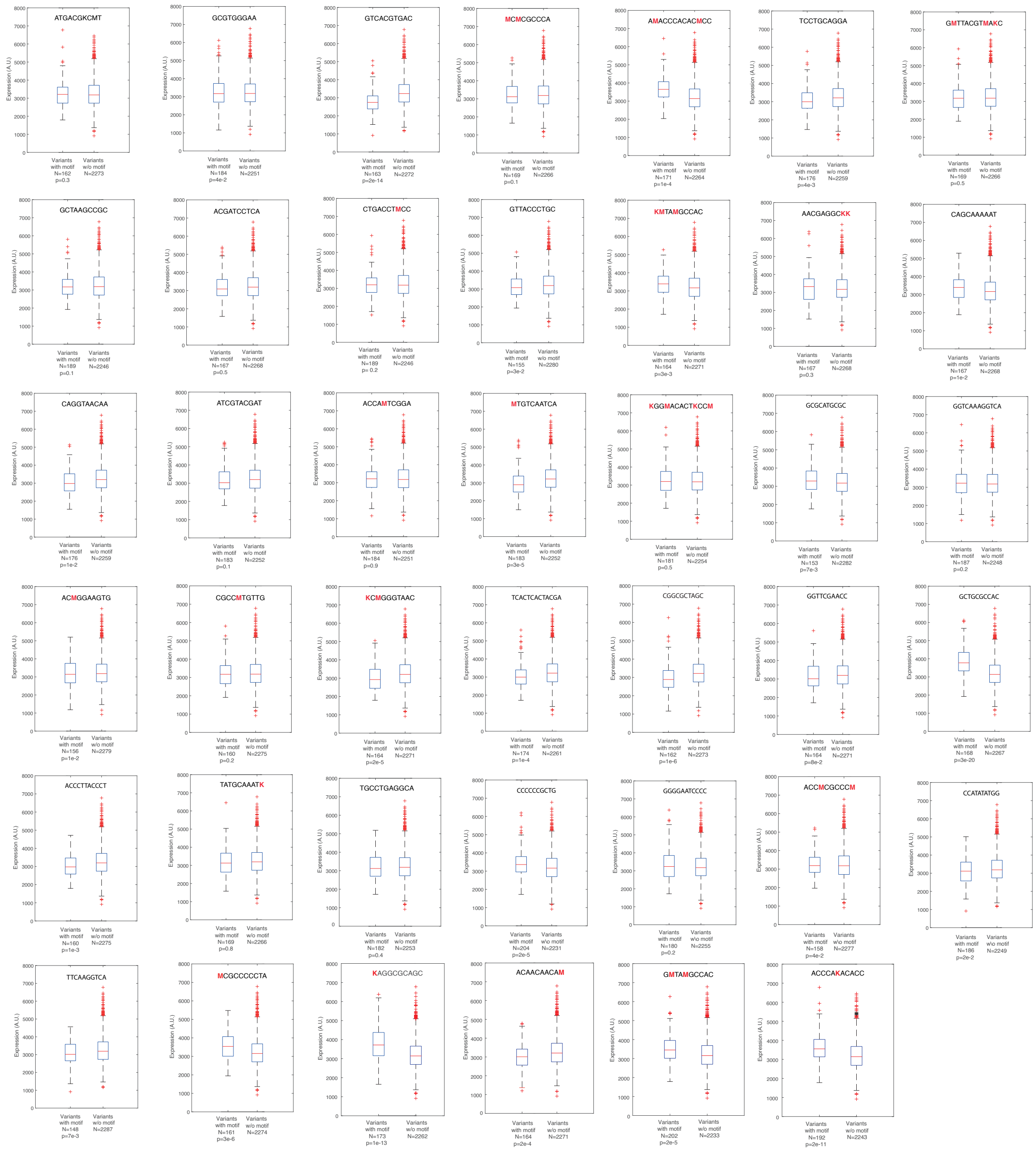

Supplementary Figure 3: The mean FL distribution of the 41 motifs used in sURS OL.

Box-plot distributions for each motif of mean FL of all variants with a specific motif (left) and variants without the motif (right). Motif that has K and/or M bases, the analysis includes all its sub-motifs. On each box, the central mark indicates the median, and the bottom and top edges of the box indicate the 25th and 75th percentiles, respectively. The value for ‘Whisker’ corresponds to ±1.5 IQR (interquartile rate) and extends to the

adjacent value, which is the most extreme data value that is not an outlier. The outliers are plotted individually as plus signs. Source data are provided as a Source Data file.

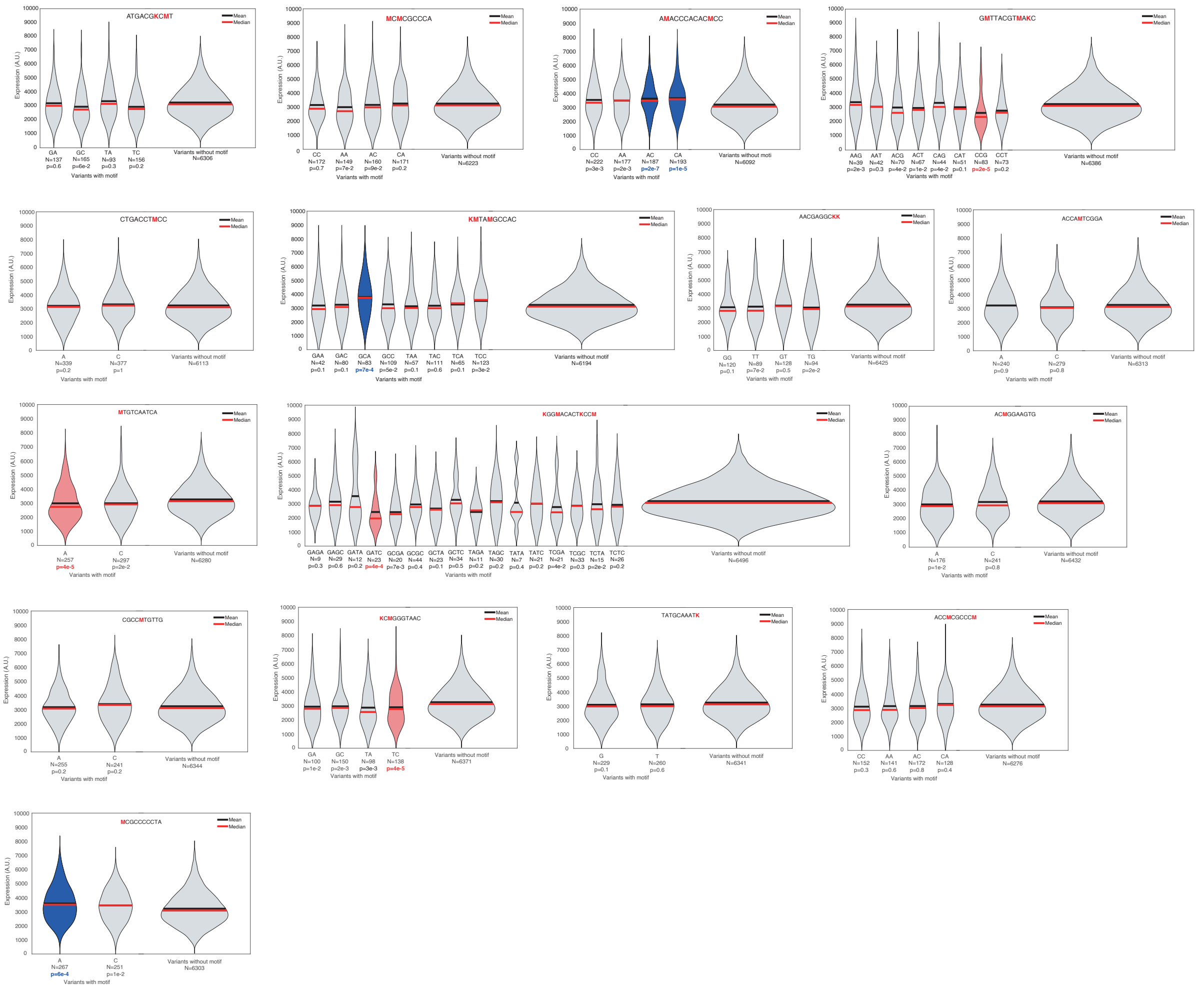

Supplementary Figure 4: Sub-motif analysis to all 20 motifs containing K and/or M mixed bases.

Violin plots for all variants containing the various sub-motifs (from 20 motifs containing K and/or M bases) as compared with all sub-variants that do not contain the sub-motif. Blue shaded violin plots are sub-motifs that were determined to be significantly activating according to mHG analysis. Red shaded violin plots are sub-motifs that were determined to be significantly repressing, based on the analysis. Source data are provided as a Source Data file.

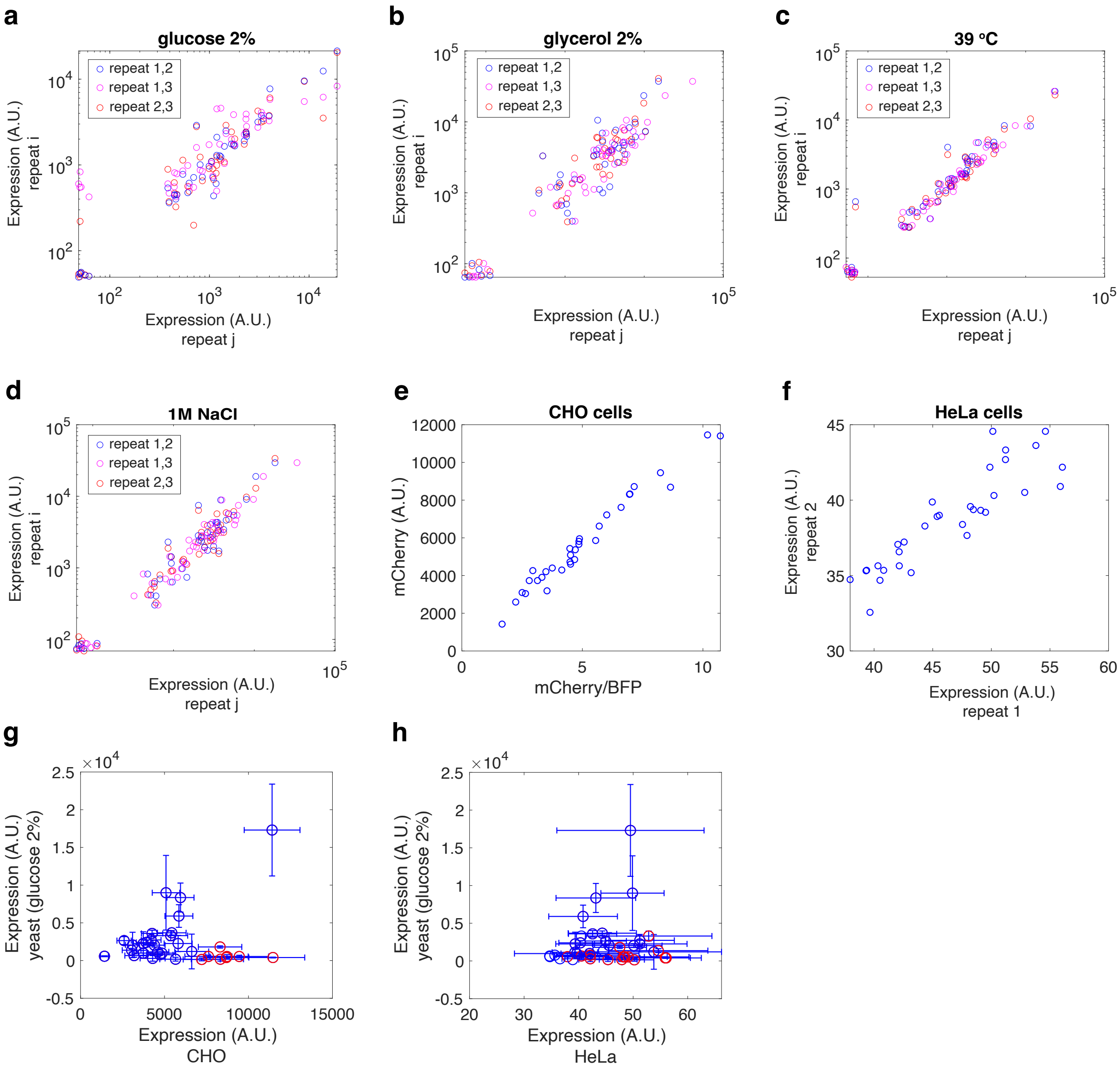

Supplementary Figure 5: Reproducibility analysis of validation sets

(a-d) Scatter plots depicting all the biological repeats for the different experimental conditions used in the Yeast experiments. (a) Glucose 2%. (b) Glycerol 2%. (c) Glucose 2% @ 39°C. (d) Glucose 2% supplemented by 1M NaCl. The biological repeats show a tight correlation for all conditions tested. (e) Comparison for CHO cells for mCherry measurements to mCherry measurements normalized by BFP – showing no effect of BFP expression on results. We used a strongly expressing BFP as a “house-keeping” gene to ensure that no false positive mCherry cells were included in the analysis. (f) Scatter plot depicting two biological repeats for the measurements carried out in HeLa cells. (g-h) The 2% glucose yeast expression data plotted as a function of CHO (h) and HeLa (h) expression data. Error-bars were computed using standard-error analysis carried out on

mean flow cytometry fluorescence measurements obtained from three or four biological repeats – depending on data set. Source data are provided as a Source Data file.

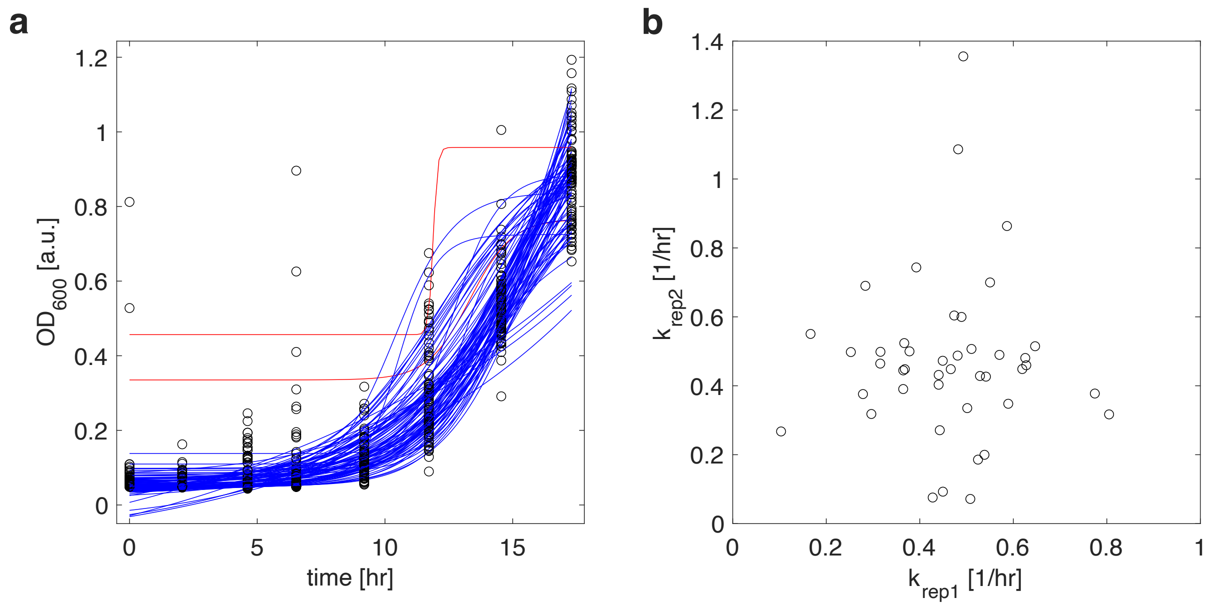

Supplementary Figure 6: Growth curve analysis of validation sets

(a) The 43 validation variants were grown in 2% glucose in duplicates and tracked for OD as a function of time (circles). For each variant the growth data are fitted (blue lines) by a classic growth curve (see Supplementary Information). (b) The rates of growth for each variant are plotted as a scatter plot pair for both repeats. Source data are provided as a Source Data file.

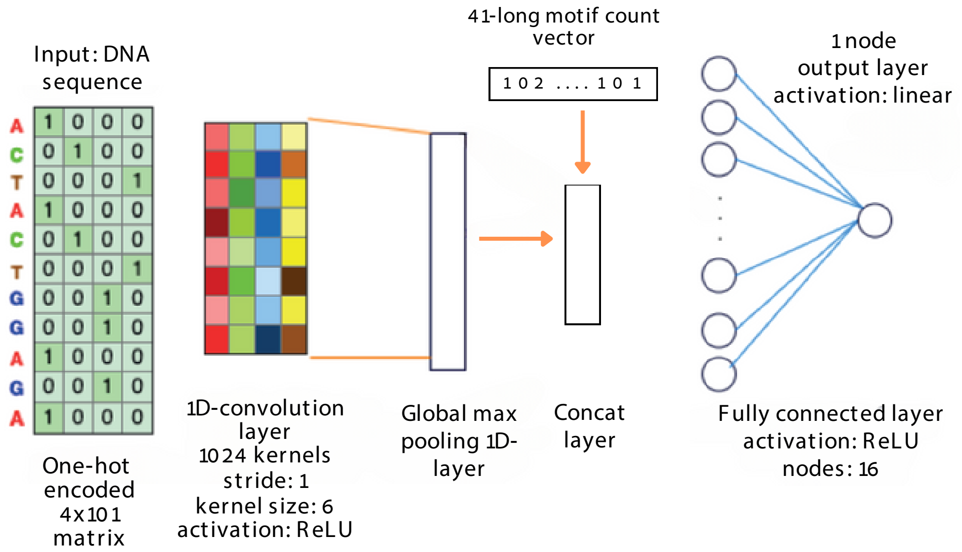

Supplementary Figure 7: MLAM model scheme

Scheme showing how the machine learning model was concatenated with the MAM model to produce the MLAM scores.

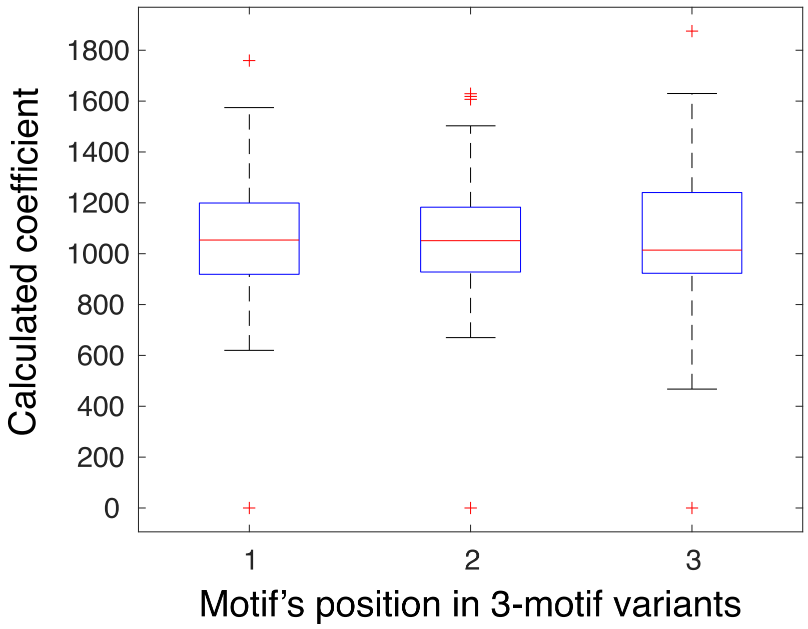

Supplementary Figure 8: Binding site motif coefficients distribution over the three positions.

We optimized 126 variables to minimize the squared error of 2435 linear equations. Each equation is a sum of variables corresponding to a specific binding site in a specific position equal to the experimental mean fluorescence level. Source data are provided as a Source Data file.
