# Supplementary material for: A universal system for boosting gene expression in eukaryotic cell-lines
Source: Nat Commun. 2024 Mar 16;15:2394. doi: 10.1038/s41467-024-46573-5 (PMC10944472; doi:10.1038/s41467-024-46573-5)
Supplement: Supplementary file 2 — Reporting Summary [file 41467_2024_46573_MOESM2_ESM.pdf]

nature portfolio

Corresponding author(s): Roee Amit

Last updated by author(s): Feb 29, 2024

Reporting Summary

Nature Portfolio wishes to improve the reproducibility of the work that we publish. This form provides structure for consistency and transparency in reporting. For further information on Nature Portfolio policies, see our Editorial Policies and the Editorial Policy Checklist.

Statistics

For all statistical analyses, confirm that the following items are present in the figure legend, table legend, main text, or Methods section.

n/a	Confirmed	
☐	☒	The exact sample size (n) for each experimental group/condition, given as a discrete number and unit of measurement
☐	☒	A statement on whether measurements were taken from distinct samples or whether the same sample was measured repeatedly
☐	☒	The statistical test(s) used AND whether they are one- or two-sided<br>*Only common tests should be described solely by name; describe more complex techniques in the Methods section.*
☒	☐	A description of all covariates tested
☐	☒	A description of any assumptions or corrections, such as tests of normality and adjustment for multiple comparisons
☐	☒	A full description of the statistical parameters including central tendency (e.g. means) or other basic estimates (e.g. regression coefficient) AND variation (e.g. standard deviation) or associated estimates of uncertainty (e.g. confidence intervals)
☐	☒	For null hypothesis testing, the test statistic (e.g. F, t, r) with confidence intervals, effect sizes, degrees of freedom and P value noted<br>*Give P values as exact values whenever suitable.*
☒	☐	For Bayesian analysis, information on the choice of priors and Markov chain Monte Carlo settings
☒	☐	For hierarchical and complex designs, identification of the appropriate level for tests and full reporting of outcomes
☐	☒	Estimates of effect sizes (e.g. Cohen's d, Pearson's r), indicating how they were calculated

Our web collection on statistics for biologists contains articles on many of the points above.

Software and code

Policy information about availability of computer code

Data collection	Flow cytometry data for the SORT-seq experiment were collected using BD FACSAria-IIIu cell sorter. For the single variant validation experiments, the data were collected using the Miltenyi Biotec MACSQuant VYB flow cytometer. The two deep sequencing runs, post SORT-seq, were conducted using the NOVAseq 6000 system and the S1 kit.
Data analysis	Samples from the flow cytometry systems were analysed using the BD FACSDiva software version 8.0.1 and the MACSQuant software, for the SORT-seq and validation experiments, respectively. Specifically, the analyses for comparison between different conditions (Glucose, glycerol, high temperature and high concentration of NaCl) were analysed by MATLAB software (R2019b).<br>NGS data were analysed by MATLAB software (R2019b).<br>The code, trained models, and processed datasets are publicly available via github.com/OrensteinLab/UniLib

For manuscripts utilizing custom algorithms or software that are central to the research but not yet described in published literature, software must be made available to editors and reviewers. We strongly encourage code deposition in a community repository (e.g. GitHub). See the Nature Portfolio guidelines for submitting code & software for further information.

Data

Policy information about availability of data

All manuscripts must include a data availability statement. This statement should provide the following information, where applicable:

- Accession codes, unique identifiers, or web links for publicly available datasets
- A description of any restrictions on data availability
- For clinical datasets or third party data, please ensure that the statement adheres to our policy

The raw sequencing files are available on the SRA website via the BioProject accession number PRJNA1061345 (https://www.ncbi.nlm.nih.gov/sra/?term=PRJNA1061345), BioSample accessions: SAMN39265674, SAMN39265675, SAMN39265676, SAMN39265677, SAMN39265678, SAMN39265679, SAMN39265680, SAMN39265681.

Research involving human participants, their data, or biological material

Policy information about studies with human participants or human data. See also policy information about sex, gender (identity/presentation), and sexual orientation and race, ethnicity and racism.

Reporting on sex and gender	N/A
Reporting on race, ethnicity, or other socially relevant groupings	N/A
Population characteristics	N/A
Recruitment	N/A
Ethics oversight	N/A

Note that full information on the approval of the study protocol must also be provided in the manuscript.

Field-specific reporting

Please select the one below that is the best fit for your research. If you are not sure, read the appropriate sections before making your selection.

[x] Life sciences   [ ] Behavioural & social sciences   [ ] Ecological, evolutionary & environmental sciences

For a reference copy of the document with all sections, see nature.com/documents/nr-reporting-summary-flat.pdf

Life sciences study design

All studies must disclose on these points even when the disclosure is negative.

Sample size	No sample-size calculation was performed in this study. When characterizing promoter function single cell measurements are considered sufficient. Consequently there was no need to set a sample size limit.
Data exclusions	No data were excluded from the study and analysis.
Replication	All validation experiments were carried out in biological triplicates and were deemed successful.
Randomization	Randomization was not relevant to this study. The study was designed to chracterize multiple promoter variants via a high-throughput approach. As a result, this was not an experiment that required a randomized sample for comparison.
Blinding	Blinding was not relevant to this study, because every sample (i.e., Library variants) was subjected to the same analysis.

Reporting for specific materials, systems and methods

We require information from authors about some types of materials, experimental systems and methods used in many studies. Here, indicate whether each material, system or method listed is relevant to your study. If you are not sure if a list item applies to your research, read the appropriate section before selecting a response.

Materials & experimental systems

n/a	Involved in the study
☒	☐ Antibodies
☐	☒ Eukaryotic cell lines
☒	☐ Palaeontology and archaeology
☒	☐ Animals and other organisms
☒	☐ Clinical data
☒	☐ Dual use research of concern
☒	☐ Plants

Methods

n/a	Involved in the study
☒	☐ ChIP-seq
☐	☒ Flow cytometry
☒	☐ MRI-based neuroimaging

Eukaryotic cell lines

Policy information about cell lines and Sex and Gender in Research

Cell line source(s)	S. cerevisiae strain W303-1A MATalpha leu2 his3 ade2 trpl ura3 was a gift from the Yoav Arava lab, Department of Biology, Technion - Israel Institute of Technology, Haifa, Israel.<br>CHO-K1/M1H strain was a gift from Oshimura lab, Graduate School of Medical Science, Tottori University, Tottori, Japan<br>HeLa strain was a gift from the Esther Meyron-Holtz lab, Department of Biotechnology and Food Engineering, Technion - Israel Institute of Technology, Haifa, Israel.
Authentication	None of the cell lines were authenticated.
Mycoplasma contamination	Cell lines were tested for mycoplasma contamination and were found negative.
Commonly misidentified lines (See ICLAC register)	No commonly misidentified cell lines were used in this study

Plants

Seed stocks	N/A
Novel plant genotypes	N/A
Authentication	N/A

Flow Cytometry

Plots

Confirm that:

☒ The axis labels state the marker and fluorochrome used (e.g. CD4-FITC).

☒ The axis scales are clearly visible. Include numbers along axes only for bottom left plot of group (a 'group' is an analysis of identical markers).

☐ All plots are contour plots with outliers or pseudocolor plots.

☒ A numerical value for number of cells or percentage (with statistics) is provided.

Methodology

Sample preparation	We grew yeast cells (W303-1A) with integrated OL in liquid media (SD+2% glucose with supplement of amino acids, without uracil) for 60 hours, at 30°C and 250rpm. At the third day, cultures' OD were measured, and 10-15 ml batches of the grown cells were centrifuged (4 minutes, 4000 rpm) and resuspended in 50 ml fresh SD+2% glucose, supplemented with tryptophan, leucine, and histidine amino acids. A day later, 10 ml cultured cells were spun down and washed with 20 ml sterile deionized water. Cells were resuspended in fresh 50 ml PBSF (50 ml PBSX1 supplemented with 50 µl BSA X100, 20 mg/ml, NEB #B9200S). We kept resuspended cells on ice until the FACS run.
Instrument	For the SORT-seq experiment: BD FACSAria-IIIu cell sorter.<br>For the single variant validation experiments: Miltenyi Biotec MACSQuant VYB flow cytometer.

Software	BD FACSFor the SORT-seq experiment: Diva software version 8.0.1 (build 2014 07 03 11 47), Firmware version 1.8 (BD FACSAria II), CST version 3.0.1, PLA version 2.0.<br>For the single variant validation experiments: MACSQuant / MATLAB software.
Cell population abundance	Post-sort fractions contained only yeast cells cells with the chosen levels of yeCitrine. 300,000 cells were collected per bin.
Gating strategy	Live and single (as opposed to doublet) cells were gated based on the FSC-SSC scatter plot

☒ Tick this box to confirm that a figure exemplifying the gating strategy is provided in the Supplementary Information.
