# Supplementary material for: A universal system for boosting gene expression in eukaryotic cell-lines
Source: Nat Commun. 2024 Mar 16;15:2394. doi: 10.1038/s41467-024-46573-5 (PMC10944472; doi:10.1038/s41467-024-46573-5)
Supplement: Supplementary file 3 — Description of Additional Supplementary Files [file 41467_2024_46573_MOESM3_ESM.pdf]

Title: Supplementary Data 1:
Description: Synthetic URSs OL design (excel file).

Title: Supplementary Data 2:
Description: Raw NGS raw data (excel file).

Title: Supplementary Data 3:
Description: MBO model predictions based on 2098 variants (excel file).

Title: Supplementary Data 4:
Description: Summary of the mHG p-values of all the 41 motifs (excel file).

Title: Supplementary Data 5:
Description: Summary of the mHG p-values of all the 20 motifs containing K and/or M (excel file).

Title: Supplementary Data 6:
Description: Variants used in validation experiments in yeast cells and CHO cells (excel file).

Title: Supplementary Data 7:
Description: Pearson correlation data for the validation sets (excel file)
